# Hospital Factors Associated with the Survival of Infants Born at Periviable Gestation: The USA National Database

**DOI:** 10.3390/children11010133

**Published:** 2024-01-22

**Authors:** Ibrahim Qattea, Amani Quatei, Mohsen A. A. Farghaly, Alshimaa Abdalla, Mohamed A. Mohamed, Hany Aly

**Affiliations:** 1 Department of Pediatrics, Nassau University Medical Center, New York, NY 11554, USA; 2 Department of Neonatalogy, Cleveland Clinic Children’s, 9500 Euclid Avenue #M31, Cleveland, OH 44195, USA; amani_katti@yahoo.com (A.Q.); shaimaa.20ap@yahoo.com (A.A.); mohamem2@ccf.org (M.A.M.); alyh@ccf.org (H.A.)

**Keywords:** NICU, preterm, survival, periviable gestation, perinatal epidemiology

## Abstract

**Background:** Reports on the survival of infants born at periviable gestation (GA of ≤24 weeks and birth weight of <500 gm) vary significantly. We aimed to determine hospital factors associated with their survival and to assess the trend for the timing of postnatal mortality in these periviable infants. **Methods:** We utilized the de-identified National Inpatient Sample (NIS) dataset of the Healthcare Cost and Utilization Project (HCUP) from the Agency for Healthcare Research and Quality (AHRQ). National data were analyzed for the years 2010–2018. Hospitals were categorized according to delivery volume, USA regions, and teaching status. **Results:** We identified 33,998,014 infants born during the study period; 76,231 infants were ≤24 weeks. Survival at birth and first 2 days of life was greatest in urban teaching hospitals in infants <24 weeks and those who completed 24 weeks, respectively. The Northeast region has the lowest survival rate. There was a significant delay in the postnatal day of mortality in periviable infants. **Conclusions:** Hospital factors are associated with increased survival rates. Improved survival in large teaching hospitals supports the need for the regionalization of care in infants born at the limits of viability. There was a significant delay in the postnatal mortality day.

## 1. Background

Resuscitation of periviable births has considerable medical and ethical challenges. The American Academy of Pediatrics (AAP) Neonatal Resuscitation Program (NRP) recommended evaluating all preterm infants of all gestational ages for their viability. If death or a high likeliness of long-term complications is certainly expected, resuscitation may be declined [1]. The American Academy of Pediatrics and the American College of Obstetricians and Gynecologists defined periviable births as those infants born at a gestational age (GA) of 20 0/7 weeks through 25 6/7 weeks [2]. Historically, infants born at a GA less than 24 weeks were considered non-viable. Consequently, they were not offered resuscitation after birth [3]; However, these infants were successfully resuscitated at many centers [4]. The reproducibility of the reported success of periviable infants has not been assessed across the nation.

The survival of periviable gestation varies significantly among various countries and medical centers within the same country. For example, Stoll et al. concluded that among 34,636 premature newborns born at 26 hospitals for the years 1993–2012, survival increased between 2009 and 2012 in infants born at 23 weeks of gestation (from 27% to 33%) and in infants born at 24 weeks (from 63% to 65%) [5]. Another study from Germany reported survival rates of 22% and 28% in infants born at a GA of 22 and 23 weeks, respectively [4]. Another study from thirteen countries within the European Union reported a wide range of survival rates for periviable infants born at 22 weeks (0–37.3%), 23 weeks (1.1–64.5%), 24 weeks (31.0–77.7%), and 25 weeks (59.1–85.7%) [6]. The variation in survival rate may be attributed to variations in the approach to perinatal care based on different guidelines [6]. Demographic and biological factors, such as prenatal care, level of neonatal care, access to and the availability of the subspecialty, and infant sex, are linked to the improved survival of premature infants [5]; however, whether these same factors are important for periviable births is unknown. Therefore, there is an unmet need to determine demographic and biological factors associated with the increased survival of infants born at a periviable GA of ≤24 weeks.

The number of periviable infants constitutes a small fraction of overall births. For example, the rate of premature deliveries at <37 weeks in the USA is around 9.63% [7]. Of them, 7% are at <28 weeks, and only <1% are born at ≤24 weeks of gestation [8]. Therefore, the experience of a specific hospital with the resuscitation of periviable infants may significantly vary according to its delivery room volume. Previous studies on rare diseases, such as hypoplastic left heart syndrome, demonstrated a significant improvement in survival when centers have greater exposure to these infants, and it was the volume of cases in the medical center rather than the surgeon experience that was associated with improved survival [9]. Whether the delivery room volume would impact survival outcomes in periviable infants is unknown.

This study utilized the National Inpatient Sample (NIS) dataset over nine years (2010 to 2018) to report the survival rate of infants born at a GA of ≤24 weeks at all hospitals in the USA and assess factors associated with increased survival. In addition, this study aimed to determine trends for a postnatal day of mortality for those who died. We hypothesized that survival is greater in urban teaching hospitals with large delivery services when compared to smaller community-based delivery hospitals, and mortality at the date of birth (day zero) has decreased over time due to a shift in practice with more active resuscitation of periviable infants.

## 2. Methods

### 2.1. Data Sources and Management

We utilized the de-identified National Inpatient Sample (NIS) dataset from the Healthcare Cost and Utilization Project (HCUP) from the Agency for Healthcare Research and Quality (AHRQ) during the years 2010–2018. HCUP contains the largest collection of hospital discharge data in the United States. The NIS dataset includes 20% of the HCUP samples weighted to represent 100% of all inpatients in the US. Each year, more than seven million cases are drawn from thousands of hospitals across the United States with various care levels (primary–tertiary), types of insurance (public or private), size of the hospital (small, medium, or large), and many other demographic and clinical characteristics. HCUP used (ICD-9-CM) diagnoses and procedure codes from 2002 to the first nine months of 2015 and (ICD-10-CM) codes from the last three months of 2015 to 2018 [10]. The NIS is designed as a random sample of all USA community hospitals from states that contribute their State Inpatient Databases to the HCUP. Data elements in the NIS are constructed in a uniform format with quality checks in place. The NIS data are available from 1988 to 2018, thereby allowing analysis of trends over time. The unweighted data contain more than 7 million hospital stays each year, whereas weighted data estimate more than 35 million hospitalizations nationally [10].

### 2.2. Study Design and Population

All infants delivered during the study period (2010–2018) were identified. Infants transferred to other facilities were not included to avoid duplication of records. Infants diagnosed with major congenital or chromosomal anomalies were excluded from the study. Infants with GAs of ≤24 weeks were selected. Survival rates were calculated for two GA categories: <24 weeks and completed 24 weeks. Survival rate within each GA category was compared in different hospital settings (urban teaching, urban non-teaching, and rural) and USA regions (Northeast, Midwest, South, and West).

Postnatal mortality during each day of the first three days (day 0, day 1, and day 2) was calculated in each GA category (<24 weeks and completed 24 weeks). It was also determined for infants with birth weights (BWs) of <500 g and for infants with a combined GA of <24 weeks and BW of <500 g. To assess the impact of the number of annual deliveries on survival, we categorized hospitals in increments of 2000 annual deliveries, thereby yielding five hospital categories with annual deliveries of ≤2000, 2001–4000, 4001–6000, 6001–8000, and >8000 deliveries/year. We compared survival rates among these five categories using chi-square test after excluding mortalities on day 0 that were presumably related to unviability. Cochran–Armitage trend test was used to assess trends for mortality in postnatal days 0, 1, and 2. Significance was considered when the *p*-value was <0.05. This study used weighted data to represent the entire US.

## 3. Results

**Figure 1:**
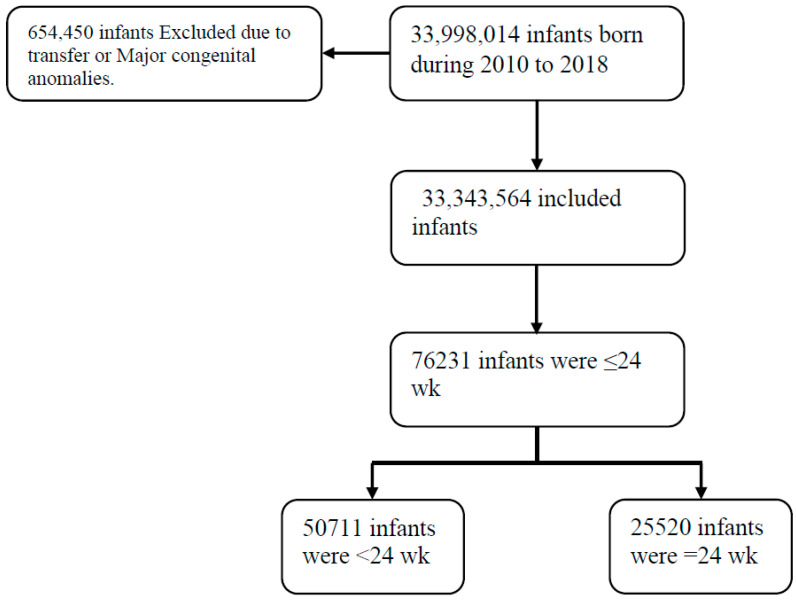
Study population.

This study identified 33,998,014 infants born during the years 2010 to 2018. A total of 654,450 infants were excluded from this study due to being transferred or having chromosomal or major congenital anomalies (Figure 1). Among the 33,343,564 included infants, 76,231 infants had GAs of ≤24 weeks; of them, 34,939 (45.8%) were females, and 22,834 (30%) were White. The number of infants at <24 weeks was 50,711, and there were 25,520 who completed 24 weeks (Figure 1).

**Figure 2:**
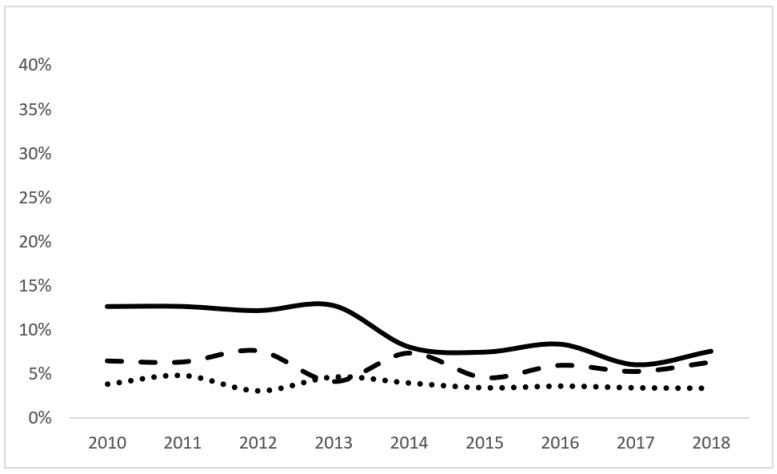
Trends in mortality for infants with completed 24-week GA based on length of stay. The solid line represents the percent of mortality of preterm babies who completed 24 weeks at day of life 0 (Z = 10.14, *p* < 0.001), the dashed line represents the percent of mortality of preterm babies who completed 24 weeks at day of life 1 (Z = 1.39, *p* 0.16), and the dotted line represents the percent of mortality of preterm babies who completed 24 weeks at day of life 2 (Z = 2.37, *p* 0.02).

Table 1 demonstrates survival rates for hospital demographics. Survival rates were significantly greater in urban teaching hospitals when compared to non-teaching or rural hospitals. The survival rate in infants with GAs of <24 weeks and infants who completed 24 weeks was greater in urban teaching hospitals. Figure 2 shows the survival rates among the four regions of the US: Northeast, Midwest, South, and West; survival rates were the lowest in the Northeast region in infants with GAs of <24 weeks (12.5%, 16.5%, 18.2%, and 14.2%, *p* < 0.001) and in infants who completed 24 weeks (55.5%, 62.2%, 61.8%, 63.7%, *p* < 0.001), respectively.

There were incremental increases in survival rates for hospital discharge in medical centers with larger delivery services (Table 2). The effect of annual delivery volume was most observed in infants with a combined GA of <24 weeks and BW of <500, wherein survival ranged from 13.7% to 22.4% in medical centers with annual deliveries of ≤2000 and >8000, respectively. Table 3 Shows the trends in mortality for infants born at less than 24-week GA based on length of stay, according to if died on day 0, died on day 1 and died on day 2, respectively. 

Postnatal mortalities in infants born at <24 weeks during the first three days were 59.1%, 31.5%, and 8.2% on days of life 0, 1, and 2, respectively. For infants with BWs of <500 g, postnatal mortalities were 66%, 36.8%, and 9.6% on days of life 0, 1, and 2, respectively. For infants with a combined GA of <24 weeks and BW of <500 g, postnatal mortalities were 76%, 59%, and 12.6% on days of life 0, 1, and 2, respectively.

**Figure 3:**
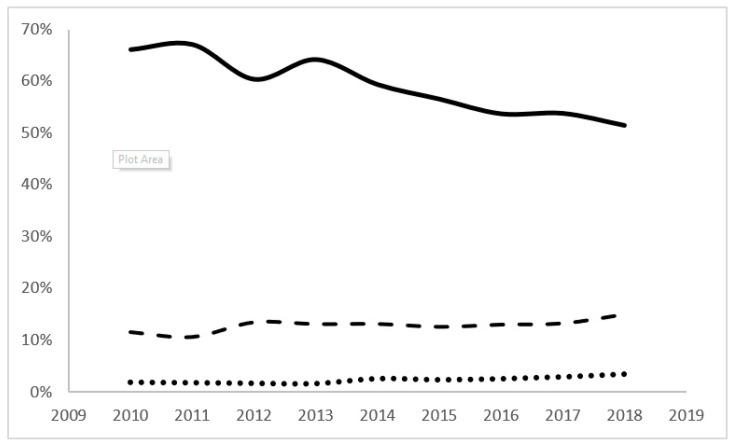
Trends in mortality for infants born at less than 24-week GA based on length of stay. The solid line represents the percent of mortality of preterm babies with <24 weeks at day of life 0 (Z = 10.30, *p* < 0.001), the dashed line represents the percent of mortality of preterm babies with <24 weeks at day of life 1 (Z = 4.03, *p* < 0.001), and the dotted line represents the percent of mortality of preterm babies with <24 weeks at day of life 2 (Z = 7.25, *p* < 0.001).

There was a significant trend for decreasing mortality on day 0 (from 66.1 to 55.5%) and day 1 (from 34% to 30.9%) and increasing mortality on day 2 (from 8.4% to 10.1%) in infants with GAs of <24 weeks. The infants born with BWs of <500 g and a combined GA of <24 weeks and BW of <500 g showed a trend for decreasing mortality on day 0 only (Figure 3).

## 4. Discussion

This study utilized data on 33,998,014 infants to report the national survival trends of periviable infants with GAs of ≤24 weeks and BWs of <500 g. Hospital factors associated with improved survival were delivery at urban teaching hospitals and hospitals with high delivery volumes. The survival of periviable infants was the lowest in the Northeast region. For infants who died, there was a significant delay in the postnatal day of mortality over the years as mortality at postnatal day 0 has decreased significantly.

The survival of periviable infants with GAs of ≤24 weeks was greater in urban teaching hospitals. This novel finding aligns with previous studies conducted on infants with very low birth weight that demonstrated improved survival, up to threefold, in tertiary care perinatal centers staffed with subspecialty teams. The lower mortality at tertiary centers is explained by the significant experience attributed to high volume, the emphasis on education and quality improvement, and the consistent implementation of updated guidelines [11,12].

The Northeast region had the lowest survival rate for periviable infants. In infants with GAs of <24 weeks, there was an almost 50% increase in survival between the Northeast and the South regions (12.5% vs. 18.2%). However, for infants who completed 24 weeks of gestation, disparities in survival rates in different regions were nominal; the West region had the highest survival rate (63.7%), whereas the Northeast region had the lowest survival rate of 55.5%. Regional survival variability is likely attributed to differences in proactive interventions and resuscitations offered to periviable infants in various USA regions. It is unclear why the Northeast region is less proactive in rescuing periviable infants, although there are possibilities to explain this phenomenon. Maternal characteristics are known to influence the decision to resuscitate periviable infants. A previous study demonstrated a regional difference in interventions for periviable infants; Midwest and South regions are more likely to administer prenatal steroids, perform cesarean delivery, and resuscitate infants at delivery when compared to Northeast and West regions [13].

Nonetheless, caregivers’ and institutions’ norms are shown to be more influential on decisions to resuscitate periviable infants [13]. Previous studies demonstrated discordance among providers regarding their preferred actions for 23 and 22 weeks of gestation deliveries [14]. Therefore, the current study illustrates the need to have system-based interventions to ensure the equity of care provided to periviable infants. Guidelines for handling and resuscitating periviable infants are required to eliminate the significant variation in practice across the nation.

There was an incremental increase in survival rates in hospitals with greater delivery volumes. In infants with GAs of <24 weeks and infants with BWs of <500 g, the survival rate was noted to increase with increasing annual deliveries up to 6000. However, for infants with a combined GA of <24 weeks and BW of <500 g, the highest survival rate was achieved in centers with annual deliveries >8000. The finding in this study supports the call for regionalization of care as referral centers with the highest delivery volumes tend to have the expertise and facilities to care for these infants. Previous studies demonstrated the positive impact of high volume on the survival of infants with different pathologies, including certain congenital heart disease and congenital diaphragmatic hernia [9,15].

The majority of mortality in periviable infants occurs within the first three days of life (day 0–day 2). Mortality in the first 24 h (day 0) constitutes the main bulk of all mortalities. This study reported a significant trend for decreasing mortality on day 0 over the years, from 66.1% in 2010 to 51.4% in 2018. The decreased mortality on days 0 and 1, despite a known increase in the resuscitation of periviable infants [16,17,18], reflects a significant improvement in experience and care provided to periviable infants. On the other hand, more non-viable infants are presently surviving through days 0 and 1, leading to significantly increased mortalities from 8.4% to 10.1% during day 2 of life. Therefore, it is wise for a caregiver to be conservative about survival estimates when counseling families during the first three days of life.

A strength of this study is that it included all deliveries in the US, thereby representing the entire nation without biases associated with the selection of major urban or academic centers [19]. This study included multiple years; consequently, the national trend for the survival of periviable infants was accurately estimated. This administrative database depends on ICD-9 and ICD-10 coding for diagnoses. Therefore, errors related to death and survival are almost impossible. One of the limitations of this study is the lack of detailed information on clinical presentation, risk factors, and interventions of periviable infants. Furthermore, the HCUP provides only an inpatient dataset; therefore, the long-term neurodevelopmental outcome of periviable infants was not reported in this study. Although stillbirths are not captured by HCUP data, as it includes only live admissions, this is out of the scope of the current study.

## 5. Conclusions

The survival rate for infants at the limit of viability has significantly increased during the period 2010–2018. The Northeast region had the lowest survival rate in the USA. In addition to the USA region, other hospital factors that influenced the survival of periviable infants were the volume of deliveries and the hospital’s teaching status. This study supports the need for regionalization of care in infants born at the limits of viability and the need to establish clear guidelines for managing periviable infants to eliminate health disparities and decrease the discrepancy in the active treatments that impact the survival of periviable infants geographically.

## Figures and Tables

**Table 1:** Factors of location of delivery for infants with <24-week GA and completed 24-week GA.

		<24-Week GA			Completed 24-Week GA		
		Total	Alive (%)	*p*-Value	Total	Alive (%)	*p*-Value
Location/teaching status of hospital	Rural	2258	75 (3.3)		345	138 (40)	
	Urban non-teaching	9352	824 (8.8)		2962	1654 (55.8)	
	Urban teaching	38,762	7171 (18.5)		22,094	13,820 (62.6)	
				<0.001			<0.001
Region of the hospital	Northeast	8740	1090 (12.5)		3584	1990 (55.5)	
	Midwest	11,677	1928 (16.5)		5409	3362 (62.2)	
	South	19,642	3576 (18.2)		11,214	6928 (61.8)	
	West	10,653	1515 (14.2)		5313	3387 (63.7)	
				<0.001			<0.001
Control/ownership of hospital	Government, nonfederal	6394	1204 (18.8)		3623	2212 (61.1)	
	Private, not-for-profit	38,506	5989 (15.6)		19,326	11,989 (62)	
	Private, investment ownership	5472	876 (16)		2452	1411 (57.5)	
				<0.001			<0.001

Data are expressed as numbers (%). Chi-square test was used for analyses.

**Table 2:** Survival of infants at less than 24-week GA and less than 500 g birth weight based on hospital delivery size.

Deliveries/Year	Survival % during Hospital Stay (Excluding Who Babies Died on Day 0)		
	<24 Weeks	<500 gm	<24 Weeks and <500 g
<2000 deliveries/year	22.0%	19.1%	13.7%
2000–4000 deliveries/year	23.5%	25.8%	17.5%
4000–6000 deliveries/year	24.7%	28%	17.6%
6000–8000 deliveries/year	26.6%	30.2%	18.1%
>8000 deliveries/year	26.9%	30%	22.4%
*p*-value	<0.001	<0.03	<0.001

**Table 3:** Trends in mortality for infants born at less than 24-week GA based on length of stay.

	Died on Day 0	Died on Day 1	Died on Day 2
2010	66.1%	11.5%	1.9%
2011	67.0%	10.6%	1.8%
2012	60.3%	13.5%	1.7%
2013	64.1%	13.1%	1.6%
2014	59.3%	13.1%	2.6%
2015	56.5%	12.6%	2.3%
2016	53.7%	13.0%	2.5%
2017	53.7%	13.3%	2.9%
2018	51.4%	15.0%	3.4%

## Data Availability

The data available at https://hcup-us.ahrq.gov/dataquerytools.jsp (accessed on 8 January 2024).
